# Status Epilepticus Treated With Vagus Nerve Stimulation in an Adult With Cortical Dysplasia: A Case Report

**DOI:** 10.7759/cureus.75964

**Published:** 2024-12-18

**Authors:** Irene Gómez-Oropeza, Valeria I Bravo-Osorno, Maria F Castelo-Pablos, Jonathan U Macías-Lopez, Karen J Camarena-Rubio, Diego Pichardo-Rojas, Sonia I Mejía-Pérez, Elma Paredes-Aragón

**Affiliations:** 1 Epilepsy Clinic, National Institute of Neurology and Neurosurgery, Mexico City, MEX; 2 Epilepsy Clinic, Instituto Nacional de Neurología y Neurocirugía Manuel Velasco Suárez, Mexico City, MEX; 3 Neurosurgical Oncology and Epilepsy Surgery, National Institute of Neurology and Neurosurgery, Mexico City, MEX

**Keywords:** case report, drug-resistant epilepsy (dre), focal cortical dysplasia, status epilepticus (se), vagus nerve stimulation

## Abstract

Status epilepticus (SE) is a neurological emergency characterized by prolonged seizures, with significant risks of neuronal injury and mortality. This case presents a 60-year-old man with drug-resistant epilepsy and a history of recurrent prolonged seizures. His seizures began in early childhood and persisted despite multiple anti-seizure medications. Notably, his semiology included the *chapeau de gendarme* sign, suggesting an involvement of the anterior cingulate cortex and frontal lobe. Brain MRI revealed a cortical dysplasia in the right inferior frontal gyrus. Given his high risk of sudden unexpected death in epilepsy (SUDEP) due to frequent generalized tonic-clonic seizures, vagus nerve stimulation (VNS) was considered an alternative therapy, with future evaluation for a surgical intervention involving lesionectomy. Following VNS implantation, his SE was arrested with standard titration, and within six months, the patient achieved seizure freedom. This case demonstrates the use of VNS in controlling SE. Further research is necessary to determine optimal protocols for VNS use in SE.

## Introduction

Status epilepticus (SE) is a condition resulting from either the failure of seizure termination mechanisms or from the initiation of mechanisms that enable abnormally prolonged seizures and can have long-term consequences including neuronal death, neuronal injury, and alteration of neuronal networks. It is characterized by prolonged seizures, operationally defined as ≥5 min of continuous seizure or two or more discrete seizures between which there is incomplete recovery of consciousness [1].

Vagus nerve stimulation (VNS) was approved for adjunctive treatment of drug-resistant epilepsy (DRE) in Europe in 1994, followed by approval in the USA three years later and in Canada shortly after [2]. VNS leads to the release of catecholamines, particularly norepinephrine. This release increases the seizure threshold and modulates thalamocortical circuits, contributing to the reduction in seizure frequency. The ability of VNS to modulate neural circuits and reduce seizure frequency offers a promising therapeutic avenue for patients who do not respond to anti-seizure medications [3].

VNS has been used for refractory SE (RSE), which persists despite second-line treatment. A recent systematic review showed that VNS can interrupt RSE and SRSE in 74% of patients [4]. The review emphasized the need for more studies to understand better the optimal use of VNS in this patient population, as the current evidence is limited and varied in quality. This case report aims to document the management of an adult patient with DRE, emphasizing the challenges of SE and the effectiveness of VNS as an alternative therapy.

## Case presentation

A 60-year-old right-handed man with a previous diagnosis of epilepsy presented to our Neurology Department. Regarding his perinatal history, he was hospitalized for one week due to neonatal hypoxia. He has controlled hypertension and no current additional comorbidities. He reports no significant family history of epilepsy.

His seizure semiology began at two years old with behavioral arrest and loss of consciousness, mirthless laughter, and subsequent generalized tonic posture. Seizures usually lasted three minutes and his postictal period was characterized by disorientation, drowsiness, and sometimes psychomotor agitation. He had approximately two seizures a day with this semiology. Seizures would happen in clusters and could last hours, with a predominance for nighttime. During these recurrent prolonged seizures, he exhibited the *chapeau de gendarme* sign (a turned-down mouth, also known as ictal pouting (IP)). It seemed like focal SE, which has a high risk for sudden unexpected death in epilepsy (SUDEP).

His seizure semiology evolved and changed at age nine, which intensified with generalized rigidity, extended arms, and fisted hands. His tonic seizures were constantly associated with periods of apnea that could last two minutes. He persisted with this semiology, experiencing one to two seizures daily and a pronounced tendency toward seizure clusters.

Throughout his 58-year history of epilepsy, he consistently experienced a high seizure frequency. He reported being treated from an early age with multiple trials of antiseizure medications, including valproic acid, carbamazepine, phenytoin, and oxcarbazepine, without achieving sustained seizure freedom. His current antiseizure medication regimen included levetiracetam 1.5 g every 12 hours, lamotrigine 250 mg every 12 hours, and clonazepam 2 mg at night.

**Figure 1:**
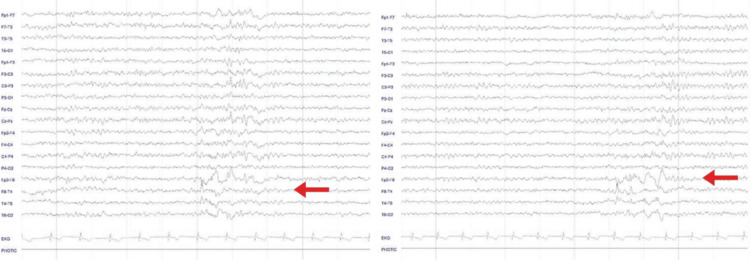
Interictal scalp EEG An epoch of a 30-minute scalp EEG with longitudinal montage showing epileptic activity over the right anterior temporal region (F8-T4-F2).

All seizures appeared to originate from the frontal lobe, with some potentially involving the anterior cingulate cortex (ACC). Previous studies included an interictal scalp electroencephalogram that showed mild right anterior temporal epileptic activity (Figure 1).

**Figure 2:**
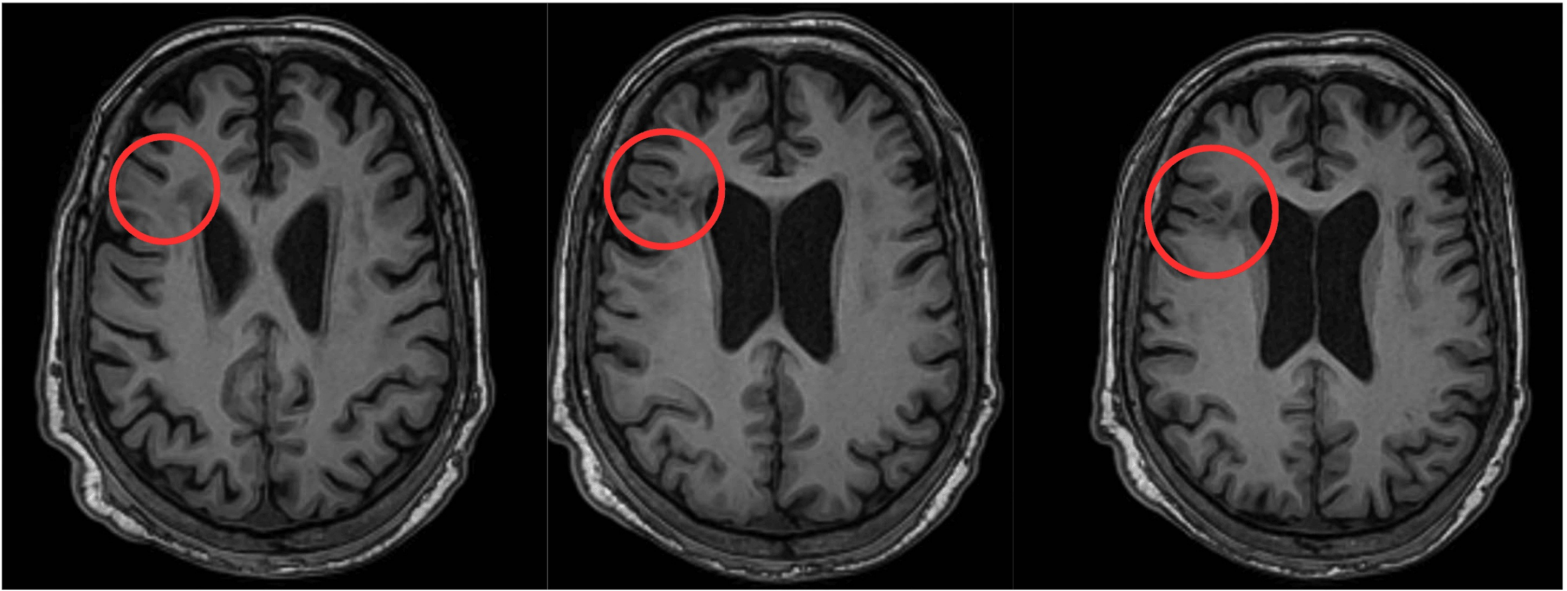
(Left to right) Brain MRI with HARNESS protocol Axial 3D BRAVO reconstruction with T1-weighted image consistent with focal cortical dysplasia (likely type 2b) in the right inferior frontal gyrus. HARNESS: Harmonized Neuroimaging of Epilepsy Structural Sequences

Brain MRI showed diffuse leukoaraiosis and bilateral hippocampal atrophy, predominately on the right. Harmonized neuroimaging of epilepsy structural sequences (HARNESS) MRI protocol showed right frontal cortical dysplasia in the inferior frontal gyrus (Figure 2).

Neuropsychological testing for anatomical correlation reported dysfunction predominantly on the right-sided frontotemporal connectivity, primarily involving medial frontal areas, predominantly the ACC. A positron emission tomography (PET)/CT scan with fluorodeoxyglucose (FDG) revealed an abnormal metabolic brain pattern compatible with generalized neuronal dysfunction. However, no distinct areas of focal hypometabolism were observed, nor was there markedly asymmetric or dominant regional hypometabolism.

With this data, the hypothesis for the epileptogenic zone was the anterior cingulate with an involvement of the primary and premotor areas, correlated to the dysplasia previously described. A decision based on the severity of the case was made, mainly focused on the SE tendency and the high risk of SUDEP related to the frequency of generalized tonic-clonic seizures. 

The case was discussed with a multidisciplinary team, and he was considered a candidate for VNS placement as acute therapy, with future evaluation for a surgical intervention involving lesionectomy. A VNS was implanted within four months, with standard titration. Interestingly, the SE was aborted immediately, even after implantation with the device turned off. He achieved seizure freedom after six months of device implantation, without initial rapid titration (Table 1). No changes in dosage or type of ASMs were made during this time. The Consensus-based Clinical Case Reporting Guideline Development (CARE) was used for this case report.

**Table 1:** VNS voltage parameters and Engel classification Generator parameters—Normal, Magnet, and AutoStim (Automatic Stimulation)—adjusted after vagus nerve stimulation (VNS) implantation (week 0), along with the Engel classification for evaluating seizure outcomes.

	Week 0	Week 2	Week 4	Week 6	Week 7	Week 9	Week 11
Voltage (mA)	-	Normal: 0.25 Autostim: 0.375 Magnet: 0.5	Normal: 0.50 Autostim: 0.75 Magnet: 1	Normal: 0.75 Autostim: 1 Magnet: 1.25	Normal: 1 Autostim: 1.25 Magnet: 1.5	Normal: 1.25 Autostim: 1.5 Magnet: 1.75	Normal: 1.5 Autostim: 1.75 Magnet: 2
Engel classification	-	1a	1a	1a	1a	1b	1a

## Discussion

This case offers an interesting instance of DRE in a 60-year-old man, with a semiology suggestive of frontal lobe epilepsy, particularly involving the ACC. A key feature in this patient’s presentation was the chapeau de gendarme sign, also known as IP, characterized by a symmetric down-turned mouth, resembling an inverted smile.

Recent studies showed evidence of the involvement of mesial frontal structures, highlighting the crucial role of ACC in IP generation. This suggests that activation occurs in the emotional insula-cingulate network rather than being confined to a single cortical area [5]. In other case series, it has been associated with frontal and temporal lobe seizures linked to cortical dysplasias [6].

The main concern for this patient was his risk factors for SUDEP. These were the following: (1) having a high frequency and prolonged duration of generalized tonic-clonic seizures (GTCS), (2) having nocturnal GTCS, and (3) not sharing a bedroom with anyone.

A case-control study showed that the history of GTCS is associated with a tenfold increased risk of SUDEP, and there is an increase if they share a household but not a bedroom [7]. In this context, the currently most preventive method is to prescribe more effective treatments that prevent focal seizures from evolving into bilateral tonic-clonic seizures, which VNS helps prevent. This study reported that VNS was associated with a 59% reduced SUDEP risk.

The clinical relevance of a case report about SE arrested by VNS in an adult patient with cortical dysplasia lies in its potential to offer an alternative therapeutic option for a life-threatening condition like SE. SE is associated with high morbidity and mortality, with outcomes depending on the condition's resistance to treatment. Poor prognostic factors for mortality in both children and adults include old age, acute etiology, and treatment resistance. Additionally, SE often leads to neurological disabilities, with a significant number of patients acquiring new disabilities post-SE [8]. VNS offers a promising approach to reducing the burden associated with SE. This case illustrates VNS as an effective therapy, even without rapid titration, highlighting its potential to improve outcomes in SE patients.

Cortical dysplasia is a common cause of DRE, and patients with this condition often experience frequent and severe seizures. SE particularly in the context of cortical dysplasia, can be resistant to conventional anti-seizure medications, necessitating the exploration of non-pharmacological options, like neuromodulation therapy.

A recent retrospective study aimed to evaluate the protective effect of VNS on the recurrence of SE [3]. The study found that patients with a history of SE had a poorer overall response to VNS when considering the response rates for all seizure types, compared to those without a history of SE. The research included 10 patients with focal cortical dysplasia. However, larger studies are necessary to determine the precise long-term effects of VNS on the risk of SE recurrence.

This case suggests that VNS can be a valuable intervention in managing SE, even with a previous history of SE. Since SE is a life-threatening condition, we considered initial treatment with VNS, followed by lesionectomy in a second surgical stage. This approach may be particularly beneficial for patients with structural brain abnormalities, such as cortical dysplasia, due to their high baseline seizure frequency.

The quality of the current data available is low due to reliance on case reports and case series, making the risk of reporting bias high. Prospective studies are needed to establish the efficacy and safety of VNS in this specific patient population.

## Conclusions

This case highlights the role of VNS in the early management of status epilepticus in a patient with DRE related to cortical dysplasia. This suggests that VNS implantation should be considered a safe and effective adjunctive treatment of SE when standard ASMs have failed, and epilepsy surgery is being evaluated. There is a lack of specific data on the outcomes of VNS without rapid titration, indicating a need for further research to establish the optimal protocols for VNS in the management of SE. Further prospective research is needed to achieve direct evidence for the efficacy of VNS in this setting.
